# Controlling the morphological, structural, and optical properties of one-dimensional PCDTBT nanotubes by template wetting

**DOI:** 10.1186/1556-276X-9-600

**Published:** 2014-11-05

**Authors:** Nor Asmaliza Bakar, Azzuliani Supangat, Khaulah Sulaiman

**Affiliations:** 1 Low Dimensional Materials Research Centre, Department of Physics, University of Malaya, Kuala Lumpur 50603, Malaysia

**Keywords:** PCDTBT, Nanotubes, Porous alumina template, Infiltration

## Abstract

In this study, the synthesis of poly [N-9′-heptadecanyl-2, 7-carbazole-alt-5, 5-(4′, 7′-di-2-thienyl-2′, 1′, 3′-benzothiadiazole)] (PCDTBT) nanotubes via a templating method is reported. PCDTBT nanotubes were successfully grown by immersing the porous alumina template into 15 mg/ml of solution concentration for 2- and 24-h periods and annealed at 50°C. Changes in morphological and optical properties between nanotubes of different infiltration times (2 and 24 h) as well as its thin films are observed. The longer infiltration time of 24 h produced nanotubes with enhanced morphological, structural, and optical properties. Nanotubes that are formed between 2 and 24 h of infiltration show enhancement in absorption, photoluminescence, and shift in Raman peak if compared to their thin films.

## Background

Conjugated polymers have been widely used in a number of technologies such as organic light emitting diodes, organic photovoltaics devices and sensors due to their favorable properties [1-4]. Optimizing the performance of such devices is rather complex due to the molecular nature of the polymer. Fabricating one-dimensional nanostructure such as nanorods, nanowires and nanotubes into the devices can enhance the photon absorption, electron transportation, and electron collection [5]. Template-assisted method is one of the simplest and cost effective methods that have been extensively used in the fabrication of polymer nanostructures [6-8]. A templating method has a great potential to produce various one-dimensional nanostructures with different favorable properties. The optical, electronic properties and polymer chain packing of the nanostructures can be altered by changing the templating technique and parameters such as varying the annealing temperature, infiltration time, spin coating rate, polymer concentration and type of solvents [8-11].

Poly [N-9′-heptadecanyl-2, 7-carbazole-alt-5, 5-(4′, 7′-di-2-thienyl-2′, 1′, 3′-benzothiadiazole)] (PCDTBT) is a promising *p*-type conjugated polymer that possesses exceptional electrical conductivity and optical properties [4,7,10,12-15]. These exceptional properties have attracted many researchers to utilize the PCDTBT thin film as an active layer in organic photovoltaic devices [10,12,13]. However, to the authors' best knowledge, no studies are available in the literature on modification of PCDTBT thin films into nanostructures. Desired PCDTBT nanostructures can be altered by controlling the infiltration time of template wetting. Characterization studies on the impact of varying the infiltration time of template wetting could provide further understanding on the growth mechanism of polymeric nanostructures. In this paper, PCDTBT nanotubes that have been prepared via template wetting are reported. These nanotubes were obtained by immersing the porous alumina template in 15 mg/ml of PCDTBT solution of two different infiltration times (2 and 24 h), prior to annealing at 50°C. This study examines the improvements in morphological, structural, and optical properties of PCDTBT nanotubes compared to their thin film counterparts.

## Methods

The commercially available PCDTBT from Luminescence Technology Corp (Taiwan, ROC) was used without further purification. The 15 mg/ml of PCDTBT solution concentration was prepared in chloroform. A template was purchased from Whatman Anodisc (Sigma-Aldrich, St. Louis, USA) with the cleaning procedures followed as in literature [16]. The template was immersed in PCDTBT solution for 2 and 24 h prior to the annealing at 50°C (1 min). The template that has been immersed in PCDTBT solution needs to be dissolved in 4M sodium hydroxide for 24 h in order to obtain the nanostructures. PCDTBT nanostructures were characterized by field emission scanning electron microscope (FESEM) (JSM 7600-F, JEOL Ltd., Tokyo, Japan), high-resolution transmission electron microscope (HRTEM) (JEM 2100-F, JEOL Ltd., Tokyo, Japan), UV-vis spectroscope (Jasco V-750, JASCO, Tokyo, Japan), photoluminescence spectroscope (PL) (Renishaw, Gloucestershire, UK), and Raman spectroscope.

## Results and discussion

### Morphological properties

**Figure 1:**
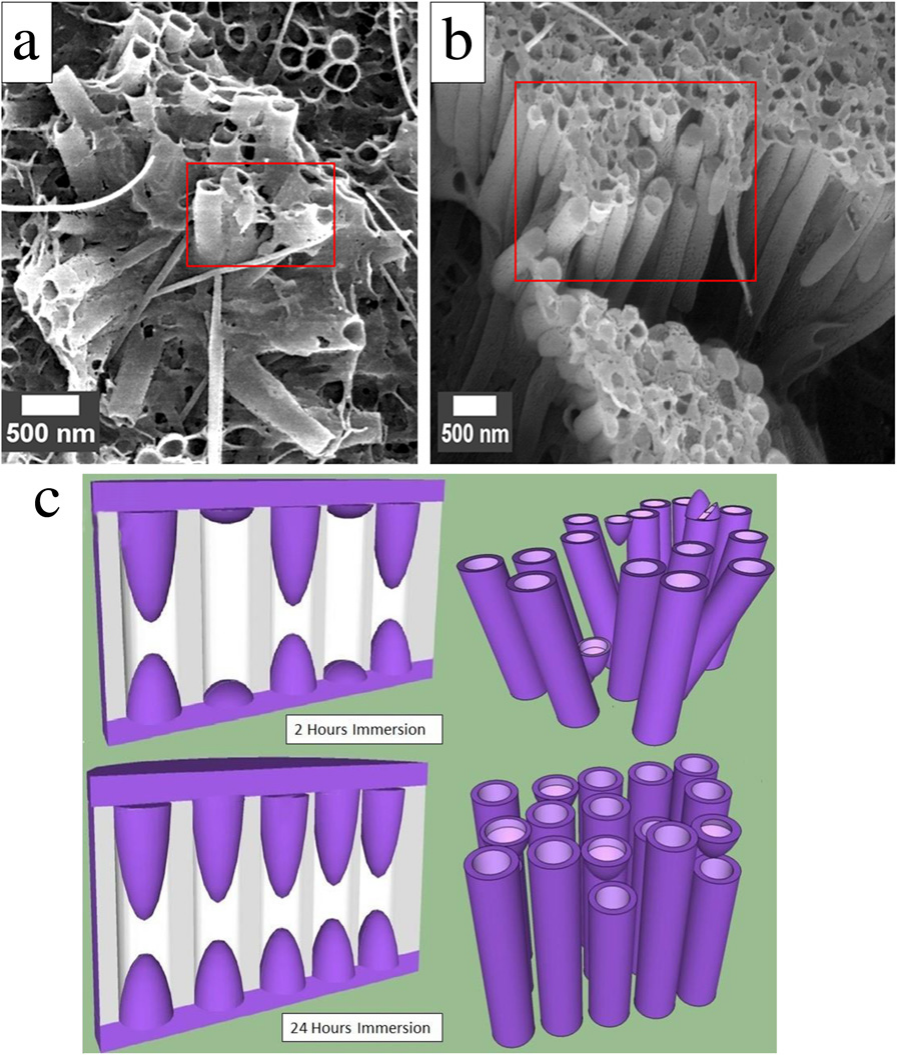
**FESEM images of PCDTBT nanotubes at different template's immersion time.** FESEM images of PCDTBT nanotubes at different template's immersion time of **(a)** 2 h and **(b)** 24 h. **(c)** Schematic diagram on the formation of PCDTBT nanotubes (alignment and densification).

Nanotubes with a wall thickness of approximately 20 nm and diameter that corresponds with the pore's diameter of approximately 200 nm were successfully fabricated via template wetting of different infiltration times (2 and 24 h). The FESEM images of PCDTBT nanotubes that were prepared from the 2 and 24 h of immersion time are shown in Figure 1a,b, respectively. The immersion time played a vital role in determining the aspect ratio and morphological distribution of the final nanotubes. In contrast to the shorter, 2 h of immersion time, longer nanotubes (with higher aspect ratio) was observed at a 24 h of immersion time. This indicates that the longer immersion time allows PCDTBT solution enough time to infiltrate deeper into the entire available template's pores. This hypothesis is also supported by the denser morphological distribution and better alignment of nanotubes observed from the 24 h of infiltration time, thus following more closely to the morphology of the original template.Fundamentally, polymer solution possesses a lower surface energy than the template's surface. Due to the higher surface energy inherited by the porous alumina template, the wetting of polymer solution onto the cavity's wall can be realized. The 2 h of infiltration time was not enough for the solution to infiltrate through the nanopores, thus resulting to a less dense and loosely-packed distribution of nanotubes. The schematic diagram in Figure 1c illustrates the different alignment, densification, and morphological distribution of PCDTBT nanotubes resulted between 2 and 24 h of immersion time. Some of the nanotubes collapsed due to the gaps created by incomplete penetration of the polymer. Depending on the application, the shorter PCDTBT nanotubes may be used for the application that required a thinner active layer such as in organic photovoltaics devices. As expected, the closer alignment with the template can be realized with the increase of the immersion time. This result suggests that different alignment, densification, and morphological distribution can be varied by the template's immersion time.

**Figure 2:**
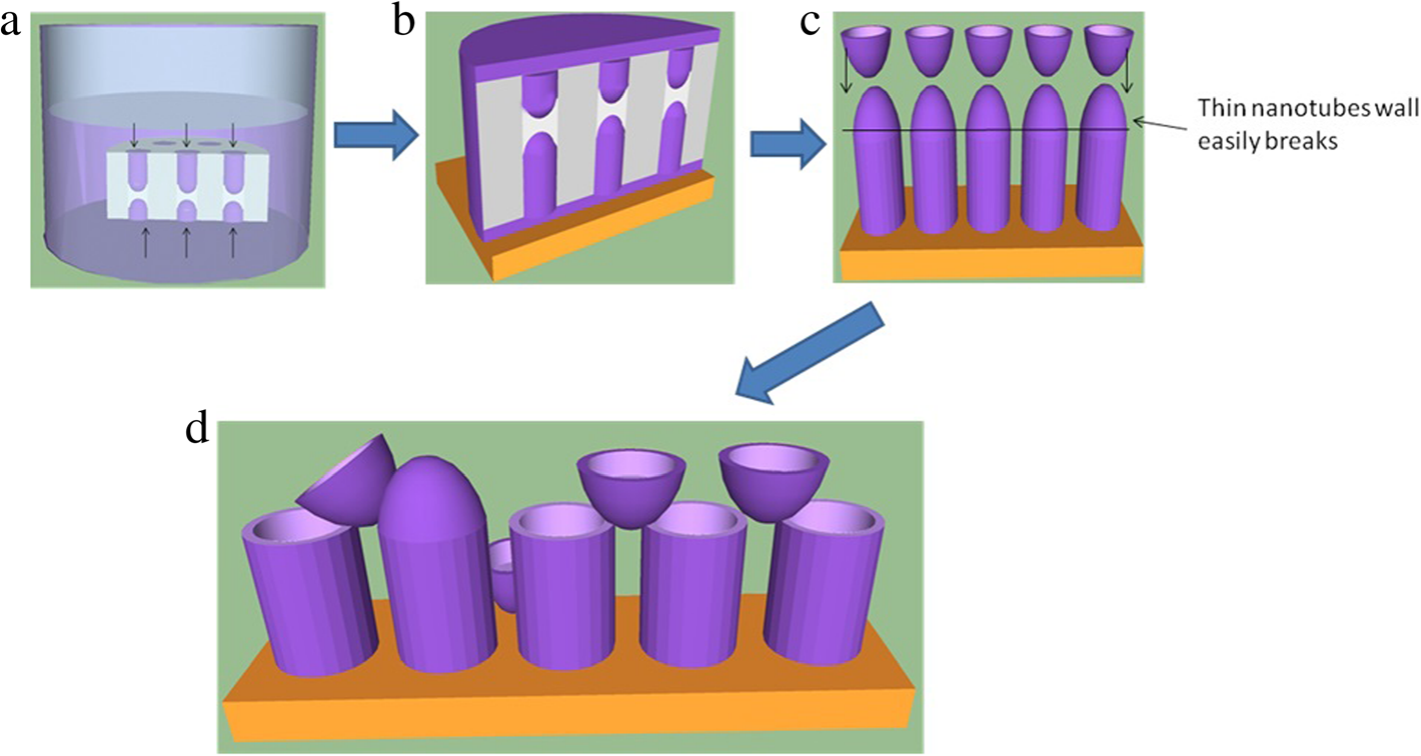
**Schematic illustrations on the formation of PCDTBT nanotubes.** Formation of PCDTBT nanotubes due to **(a)** Capillary and gravitational force of PCDTBT solution inside nanopores and **(b)** wetting and complete filling of the nanopores. **(c)** Formation of convex meniscuses and **(d)** PCDTBT nanotubes of broken meniscuses.

**Figure 3:**
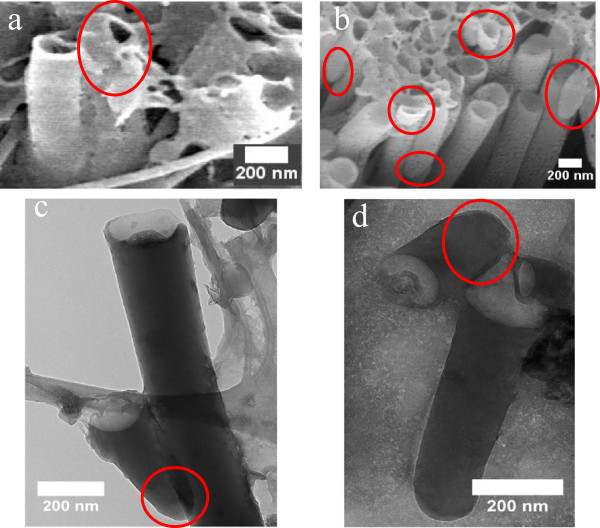
**FESEM images of PCDTBT nanotubes of (a) 2 h and (b) 24 h of immersion time.** HRTEM images of PCDTBT nanotubes of **(c)** 2 h and **(d)** 24 h of immersion time. Circles in red show the convex meniscuses.

The proposed formation of PCDTBT nanotubes is illustrated in Figure 2a,b,c,d. The PCDTBT solution infiltrates into the template by two wetting phenomena namely (i) capillary force (repulsion) which cause the solution to rise from the bottom of the template and (ii) with the assistance of gravity that contributes to the flow of solution from the top of the template into the nanopore (Figure 2a). When a solution is brought into contact with a substrate of high surface energy, the solution will spread and form a thin film. Once the template is immersed, the solution will infiltrate to a certain extent and cover the template's pore walls. Wetting and complete filling of the template's pore occur consecutively (Figure 2b). Wetting of pore walls occurs prior to the complete filling due to the stronger adhesive forces between the PCDTBT molecules and the pore wall surface rather than the cohesive forces. Rapid evaporation of solvent has inhibited the polymer solution from completely filling the template's pore which then produces nanotubes instead of nanorods [17]. During the evaporation of solvent, two convex meniscuses were formed. Once the weight of the solution is balanced by the surface tension, the solution will stop flowing from the top and cause saturation at the end of the flowing, which causes a convex meniscus (Figure 2c). Capillary repulsion causes the bottom part of convex meniscus to form. As shown in Figure 2d, the remaining PCDTBT nanostructures with the broken cap are obtained after the template's dissolution. Nanotubes that were formed at the upper part of the template will fall onto the other nanotubes that were formed in the bottom part of the template.FESEM and HRTEM images shown in Figure 3a,b,c,d gave evidence to the proposed formation of PCDTBT nanotubes caused by capillary and gravitational forces. Convex meniscuses can be observed at the tip of nanotubes (circle in red) with some of them are broken cap (Figure 3a,b). The closed end nanotubes are easily broken during the FESEM and TEM samples preparation due to the thin wall thickness of approximately 20 nm. The broken meniscus is supported by the HRTEM images shown in Figure 3c,d of 2 and 24 h of immersion time, respectively.

### Optical properties

**Figure 4:**
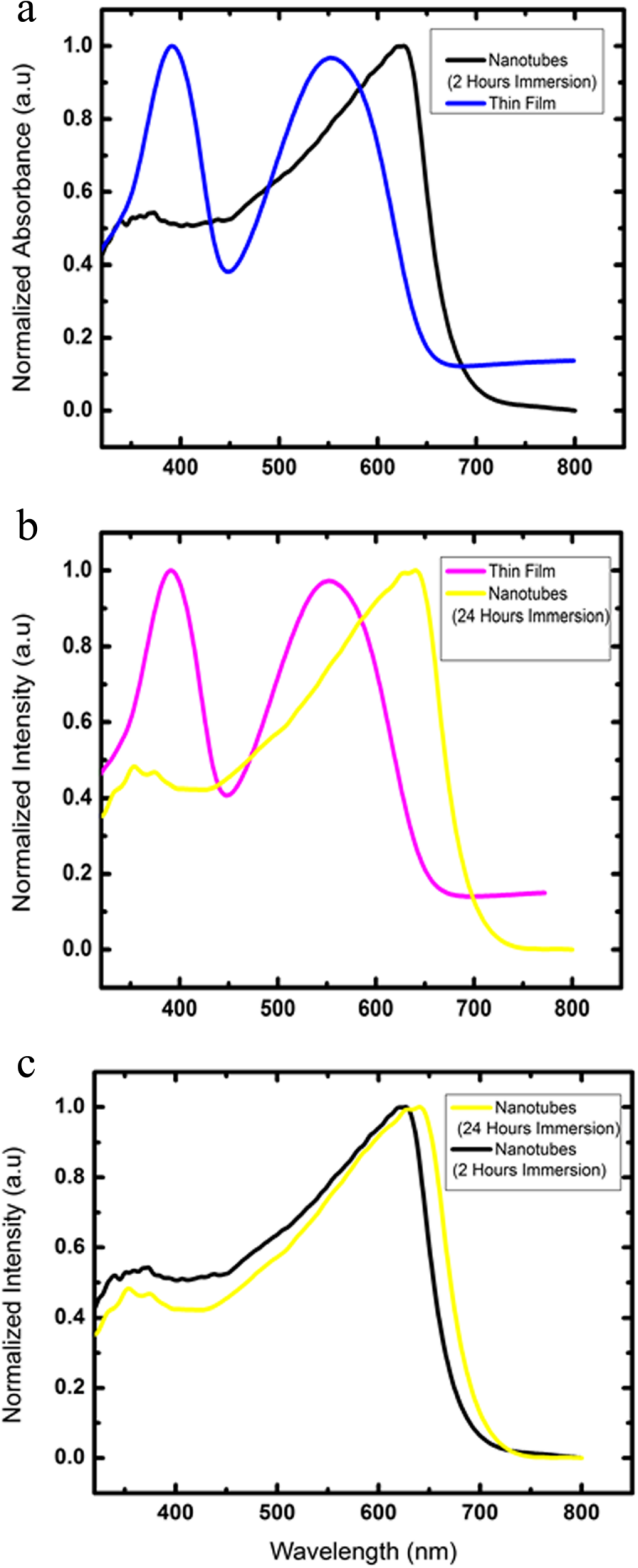
**UV-vis absorption spectra.** Comparison of UV-vis absorption spectra of **(a)** PCDTBT nanotubes of 2 h of immersion time and thin film, **(b)** PCDTBT nanotubes of 24 h of immersion time and thin film, and **(c)** PCDTBT nanotubes of 2 and 24 h of immersion time.

**Figure 5:**
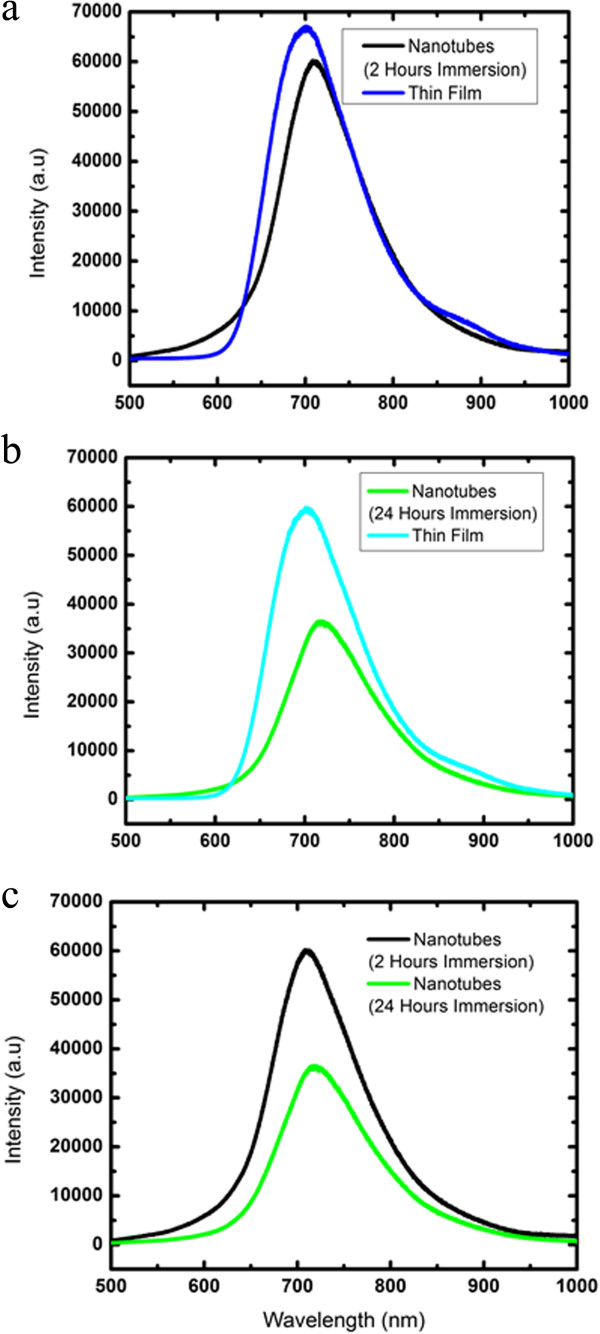
**Photoluminescence spectra.** Comparison of PL spectra of **(a)** PCDTBT nanotubes of 2 h of immersion time and thin film, **(b)** PCDTBT nanotubes of 24 h of immersion time and thin film, and **(c)** PCDTBT nanotubes of 2 and 24 h of immersion time.

The UV-vis absorption and photoluminescence (PL) spectra of PCDTBT nanotubes prepared between 2 and 24 h of immersion time are shown in Figures 4 and 5, respectively. The UV-vis absorption spectra clearly show the two absorption bands at UV and visible region which represent the donor carbazole (Cz) and acceptor dithienylbenzothiadiazole (DTBT), respectively [4]. Wider band absorption at longer wavelength could be observed for both the nanotubes prepared between 2 and 24 h of immersion time if compared to their thin films. This is an indication of better optical properties garnered by PCDTBT nanotubes in terms of light absorption. The main absorption peak of PCDTBT nanotubes is located at approximately 627 and 640 nm for 2 and 24 h of immersion time, respectively, and possesses a significant red-shift of about 75 and 88 nm as compared to their thin films. This could be attributed to the highly aggregated configuration in the polymer chain system of PCDTBT nanotubes. In the highly aggregated configuration, the polymer chains may align themselves and increase the conjugation length [18]. Aligned polymer chains provide a route for the efficient charge transfer (π to π*) between the conjugated main chains (DTBT) and carbazole (Cz) units. Modification in the molecular structure of nanotubes such as aggregation and chain alignment may improve the optical properties by enhancing the interchain and intrachain interaction. Dissimilar morphological distributions of the nanotubes give the distinct optical properties as indicated from the red-shift of about 13 nm by the nanotubes prepared for 24 h of immersion time. This is unlikely to happen to the PCDTBT nanotubes synthesized for 2 h of immersion time.

The PL spectra of PCDTBT nanotubes and thin films are shown in Figure 5a,b,c. The emission of the Cz segment was completely quenched for all the spectra with the dominance of the emissions due to the DTBT unit indicating an efficient energy transfer from Cz segment to the DTBT [14]. The quenching of photoluminescence intensity and red-shift of the PL spectra presented by both of the nanotubes prepared between 2 and 24 h of immersion time is well correlated with their UV-vis absorption spectra. The quenching of photoluminescence intensity lowered the luminescence quantum yield, increased the photon absorption, and thus magnified the optical devices performance [19].

### Structural properties

**Figure 6:**
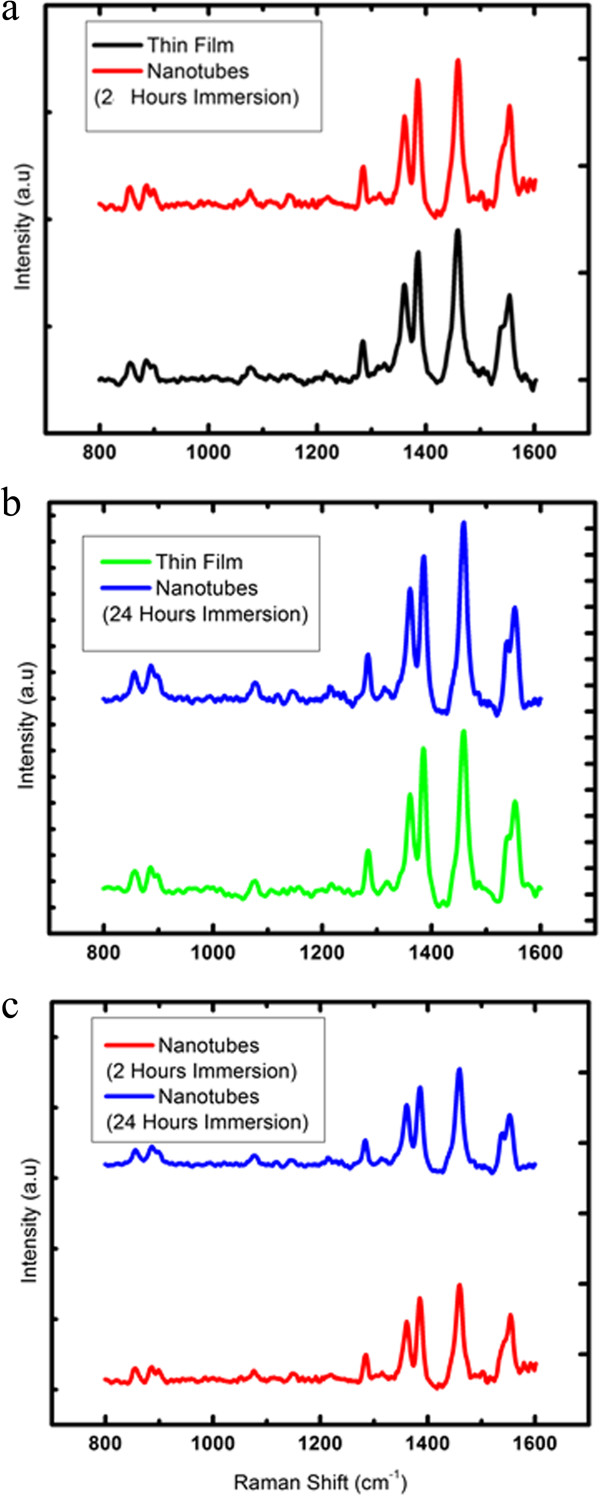
**Raman spectra.** Comparison of Raman spectra of **(a)** PCDTBT nanotubes of 2 h of immersion time and thin film, **(b)** PCDTBT nanotubes of 24 h of immersion time and thin film, and **(c)** PCDTBT nanotubes of 2 and 24 h of immersion time.

The Raman spectra and Raman peak position of PCDTBT thin films and nanotubes (2 and 24 h) are shown in Figure 6 and Table 1. Similar absorption peaks at 856, 886, and 1,284 cm^-1^ which assign for C-H in plane bending modes are observed for nanotubes and thin films. PCDTBT nanotubes prepared from 2 h of infiltration time have experienced 2 cm^-1^ of downward shift at the band that was assigned for C-S deformation. A band of C-H deformation has shown a downward shift of about approximately 3 cm^-1^ for PCDTBT nanotubes that were obtained from 24 h of immersion time, which has been previously reported elsewhere for PCDTBT nanostructures [7]. At the band assigned for C-C stretching of Cz segment (1,361 cm^-1^), no changes in the Raman shift were recorded by the PCDTBT nanotubes and thin films. However, a downward and upward shift occurred at the band of C-C stretching of the DTBT unit for PCDTBT nanotubes [4]. No changes in the Raman shift were observed between the PCDTBT nanotubes and thin films for the broad DTBT ring stretch located at 1,460 and 1,554 cm^-1^[4].

**Table 1:** Raman peak position of PCDTBT nanotubes and thin films

**Raman shift (cm**^**-1**^**)**						
**2 h of immersion**		**24 h of immersion**		**Nanotubes**		**Assignments**
**Thin film**	**Nanotubes**	**Thin film**	**Nanotubes**	**2 h**	**24 h**	
856	856	856	856	856	856	C-H bending
886	886	886	886	886	886	C-H bending
1,078	1,076	1,076	1,076	1,076	1,076	C-S deformation
1,216	1,219	1,217	1,215	1,219	1,215	C-H deformation
1,284	1,284	1,284	1,284	1,284	1,284	C-H bending
1,361	1,361	1,361	1,361	1,361	1,361	C-C stretching
1,386	1,385	1,385	1,386	1,385	1,386	C-C stretching
1,460	1,460	1,460	1,460	1,460	1,460	Ring stretch
1,554	1,554	1,553	1,553	1,554	1,553	Ring stretch

## Conclusions

In this work, PCDTBT nanotubes have been synthesized by a template wetting of a porous alumina template. Infiltration time and wetting behavior have shown to play a significant role in controlling the properties of PCDTBT nanotubes. The longer immersion time produced PCDTBT nanotubes that have more enhanced morphological, structural, and optical properties over their thin films.

## Abbreviations

FESEM: field emission scanning electron microscopy; HRTEM: high resolution transmission electron microscopy; NaOH: sodium hydroxide; PCDTBT: poly [N-9′-heptadecanyl-2, 7-carbazole-alt-5, 5-(4′, 7′-di-2-thienyl-2′, 1′, 3′-benzothiadiazole)]; PL: photoluminescence.

## Competing interests

The authors declare that they have no competing interests.

## Authors’ contributions

NAB carried out the experiment, participated in the sequence alignment, and drafted the manuscript. AS participated in the design of the study, performed the analysis, and helped draft the manuscript. KS conceived of the study and helped draft the manuscript. All authors read and approved the final manuscript.

## Authors' information

NAB is currently doing his Ph.D. at the University of Malaya. AS and KS are senior lecturers at the Department of Physics, University of Malaya. AS's and KS's research interests include the synthesis of nanostructured materials via template-assisted method and applications in organic electronic devices such as sensors and photovoltaic cells.

## References

[B1] Lek JY Lam YM Niziol J Marzec M Understanding polycarbazole-based polymer: CdSe hybrid solar cells Nanotechnology 2012 23 31 315401 10.1088/0957-4484/23/31/315401 22796943

[B2] Chen H-Y Hou J Zhang S Liang Y Yang G Yang Y Yu L Wu Y Li G Polymer solar cells with enhanced open-circuit voltage and efficiency Nature Photonics 2009 3 11 649 653 10.1038/nphoton.2009.192

[B3] Moon JS Jo J Heeger AJ Nanomorphology of PCDTBT:PC70BM bulk heterojunction solar cells Advanced Energy Materials 2012 2 3 304 308 10.1002/aenm.201100667

[B4] Jha P Koiry SP Saxena V Veerender P Gusain A Chauhan AK Debnath AK Aswal DK Gupta SK Air-stability and bending properties of flexible organic field-effect transistors based on poly[N-9′-heptadecanyl-2,7-carbazole-alt-5,5-(4′,7′-di-2-thienyl-2′,1′,3′-benzothiadiazole)] Org Electron 2013 14 10 2635 2644 10.1016/j.orgel.2013.06.031

[B5] Yu K Chen J Enhancing Solar Cell Efficiencies through 1-D Nanostructures Nanoscale Res Lett 2008 4 1 1 10

[B6] Chi MH Kao YH Wei TH Lee CW Chen JT Curved polymer nanodiscs by wetting nanopores of anodic aluminum oxide templates with polymer nanospheres Nanoscale 2014 6 3 1340 1346 10.1039/c3nr04431a 24336801

[B7] Bakar NA Supangat A Sulaiman K Elaboration of PCDTBT nanorods and nanoflowers for augmented morphological and optical properties Mater Lett 2014 131 27 30

[B8] Fakir MS Supangat A Sulaiman K Templated growth of PFO-DBT nanorod bundles by spin coating: effect of spin coating rate on the morphological, structural, and optical properties Nanoscale Res Lett 2014 9 1 225 10.1186/1556-276X-9-225 24872806 PMC4019364

[B9] Al-Kaysi RO Ghaddar TH Guirado G Fabrication of one-dimensional organic nanostructures using anodic aluminum oxide templates Journal of Nanomaterials 2009 2009 1 14

[B10] Park SH Roy A Beaupré S Cho S Coates N Moon JS Moses D Leclerc M Lee K Heeger AJ Bulk heterojunction solar cells with internal quantum efficiency approaching 100% Nature Photonics 2009 3 5 297 302 10.1038/nphoton.2009.69

[B11] Kokonou M Ioannou G Rebholz C Doumanidis CC Polymeric nanowires and nanopillars fabricated by template wetting Journal of Nanoparticle Research 2013 15 4 1 12

[B12] Clarke TM Peet J Nattestad A Drolet N Dennler G Lungenschmied C Leclerc M Mozer AJ Charge carrier mobility, bimolecular recombination and trapping in polycarbazole copolymer:fullerene (PCDTBT:PCBM) bulk heterojunction solar cells Org Electron 2012 13 11 2639 2646 10.1016/j.orgel.2012.07.037

[B13] Kannappan S Palanisamy K Tatsugi J Shin P-K Ochiai S Fabrication and characterizations of PCDTBT: PC71BM bulk heterojunction solar cell using air brush coating method J Mater Sci 2012 48 6 2308 2317

[B14] R-s L W-j Z Du B Yang W Hou Q Shi W Zhang Y Cao Y Novel red light-emitting polymers based on 2,7-carbazole and thiophene derivatives Chin J Polym Sci 2008 26 2 231 240 10.1142/S025676790800287X

[B15] Reish ME Nam S Lee W Woo HY Gordon KC A spectroscopic and DFT study of the electronic properties of carbazole-based D - A type copolymers J Phys Chem 2012 116 21255 21266

[B16] Kamarundzaman A Fakir MS Supangat A Sulaiman K Zulfiqar H Morphological and optical properties of hierarchical tubular VOPcPhO nanoflowers Mater Lett 2013 111 13 16

[B17] Steinhart M Wendorff JH Greiner A Wehrspohn RB Nielsch K Schilling J Choi J Gosele U Polymer nanotubes by wetting of ordered porous templates Science 2002 296 1997 10.1126/science.1071210 12065828

[B18] Schwartz BJ Nguyen T-Q Wu J Tolbert SH Interchain and intrachain exciton transport in conjugated polymers: ultrafast studies of energy migration in aligned MEH-PPV/mesoporous silica composites Synth Met 2001 116 35 40 10.1016/S0379-6779(00)00510-5

[B19] Schwartz BJ Conjugated polymers as molecular materials: how chain conformation and film morphology influence energy transfer and interchain interactions Annu Rev Phys Chem 2003 54 141 172 10.1146/annurev.physchem.54.011002.103811 12524429

